# A Shelf-Life Assessment of Sterilized Surgical Instruments Stored Under Controlled Conditions: A Comparative Study of a Single vs. Double Self-Sealing Pouch

**DOI:** 10.3390/vetsci12060564

**Published:** 2025-06-09

**Authors:** Stefano Cavalli, Chiara Caterino, Francesca Paola Nocera, Francesca Pizzano, Rossana Schena, Federica Aragosa, Sinem Arslan, Giovanni Della Valle, Luisa De Martino, Gerardo Fatone

**Affiliations:** Department of Veterinary Medicine and Animal Production, University of Naples Federico II, Via F. Delpino 1, 80137 Naples, Italy; stefano.cavalli@unina.it (S.C.); chiara.caterino@unina.it (C.C.); francesca.pizzano@unina.it (F.P.); rossana.schena@unina.it (R.S.); federica.aragosa@unina.it (F.A.); sinem.arslan@unina.it (S.A.); giovanni.dellavalle@unina.it (G.D.V.); luisa.demartino@unina.it (L.D.M.); fatone@unina.it (G.F.)

**Keywords:** surgical-site infections, sterilization process, sterile packaging

## Abstract

(1) Background: postoperative surgical-site infections are a significant complication in small-animal surgical procedures, with detected rates ranging from 0.8% to 18.1%, depending on the type of surgery. The sterilization process of surgical instruments is a crucial factor in infection control, but the shelf life of surgical instruments and the best packaging method are not precisely defined due to the multiple variables that influence them. This study aimed to assess the shelf life of surgical instruments stored under controlled environmental conditions in a veterinary hospital and compare two packaging methods: using a self-sealing single pouch versus a self-sealing double pouch. (2) Methods: a sample of 400 non-sterile screws was divided into three groups: Group 1 (175 screws in single pouches), Group 2 (175 screws in double pouches), and Group 3 (50 non-sterile screws as a control group to verify the microbial culture efficiency). Microbiological tests were conducted at 1, 7, 15, 30, 60, 182, and 390 days post-sterilization. (3) Results: no bacterial growth was detected on screws packaged in single and double pouches up to 182 days. However, after 390 days, bacterial growth was observed in one screw packaged in a single pouch. In Group 3, only two screws turned out to be bacteriologically positive. (4) Conclusions: no statistical difference was found between the two groups; however, the detection of a single positive screw in the single-pouch group raised a potential clinical consideration, suggesting the need for further studies based on events and time.

## Simple Summary

Postoperative infections are a common concern in small-animal surgery, and the proper sterilization of surgical instruments is essential to prevent them. This study evaluated the shelf life of sterile surgical screws stored under controlled environmental conditions at the Veterinary Teaching Hospital of Naples. We compared two packaging methods, namely single and double self-sealing pouches, over 390 days. While no bacterial growth was detected in either group up to 182 days, after 390 days, one screw in the single-pouch group showed bacterial contamination. While current guidelines focus on event-related contamination, this outcome reveals a low-level, time-dependent threat to sterility. These findings emphasize the need for future time-based studies and further evaluation of packaging methods to ensure sterility.

## 1. Introduction

Postoperative surgical-site infections (SSIs) represent one of the most frequent postoperative complications in small-animal surgeries, contributing significantly to patient morbidity, mortality, and extended hospital stays, with reported rates ranging from 0.8% to 18.1%, depending significantly on the type of procedure [1,2].

The SSIs cannot be eliminated, but preventive strategies represent the most economical and effective means of reducing their impact [3].

A crucial aspect of SSI prevention is the correct sterilization of surgical instruments. Ensuring that all materials in the operating room are sterile is vital for patient safety, as even minor lapses in sterilization can increase the risk of infection [4].

Sterilizing surgical instruments involves multiple steps, including cleaning, decontamination, and packaging [5]; while numerous protocols exist for sterilization, questions remain about how long surgical materials can be stored while maintaining sterility and which packaging method is most effective.

Accepted recommendations emphasize that sterility is event-related, not strictly time-related [6,7], meaning that sterile items could remain usable until a specific event compromises their integrity. However, when expiration dates are assigned, any expired items should be avoided [6]. A clear gap exists in the availability of controlled, long-term studies that reflect the specific environmental and procedural conditions encountered in veterinary hospitals.

The sterile packaging guidelines include only the recommendation and conditional recommendation levels, and there are no regulatory requirements or situations where the evidence suggests that no recommendation can be made [8]. For these reasons, it is of fundamental importance that every hospital periodically controls its sterilization methods.

Numerous factors influence the maintenance of surgical-material sterility; however, minimizing exposure to moisture, dust, sunlight, handling, and temperature or humidity effectively reduces the risk of contamination and degradation of the sterilized items [9]. Microbial and fungal contamination of surgical instruments poses a serious risk in clinical environments. Organisms such as *Candida* spp., *Aspergillus* spp., and bacteria including *Citrobacter* spp., *Bacillus cereus*, and *Staphylococcus* spp. can survive on surfaces and are difficult to eliminate, especially when sterilization or disinfection protocols fail [10]. This contamination can lead to postoperative bacterial or fungal infections, as many pathogenic microbes are capable of forming biofilms on the surfaces of biomedical devices [11]. It is well-established that various surface types, such as cellulose, polymers, or nylon, can serve as physical carriers of microbial contamination, regardless of the habitat [12]. Technological advancements in recent decades have introduced numerous new synthetic materials, along with the identification of microorganisms capable of biodegrading them, such as *Bacillus cereus* [13].

In recent years, many studies have focused on the packaging and storage methods for surgical instruments [14,15,16], but the most significant difficulty lies in replicating a method: every hospital operates under unique conditions, and differences in climate, cleanliness, light exposure, transportation, and storage all affect the outcomes. For this reason, the evaluation of sterilization protocols under veterinary hospital conditions is essential, where infrastructure and environmental parameters may significantly influence the maintenance of sterility. Institution-specific investigations can provide valuable data to support or adapt existing human hospital recommendations to the veterinary context.

The present study aimed to assess the shelf life of surgical instruments stored under controlled environmental conditions detected for this study at the Veterinary Teaching Hospital (VTH) of Naples. Tracking factors such as temperature, humidity, light exposure, storage conditions, and handling frequency enable the evaluation of surgical instruments’ shelf life and the effectiveness of single- versus double-pouch packaging in maintaining sterility over time. Achieving these objectives ensures the sterility of surgical materials, minimizes packaging costs, promotes environmental sustainability, and upholds patient safety.

## 2. Materials and Methods

A total of 400 non-sterile surgical screws were used for this study. A sterile processing technician was responsible for packaging the screws. The screws were divided into three groups: Group 1, containing 175 screws packaged individually in a single sterilization self-sealing paper/plastic pouch; Group 2, containing 175 screws packaged in a double sterilization self-sealing paper/plastic pouch; and Group 3, as the control group, containing 50 screws. The control group served to test the effectiveness of the microbial culture system chosen for the study. The control screws were washed and packaged identically to Group 1 but were left unsterilized.

In the double-pouch packaging (Group 2), the pouches were placed with plastic facing plastic, and paper facing paper. The sample size was calculated using a power analysis with a significance level of 0.05, power level of 0.80, and row value (effect size) of 0.25.

### 2.1. Pouch Specifications

Six hundred non-sterile self-sealing flat pouches, sized 90 mm × 260 mm, were used (REINOX GmbH, Düsseldorf, Germany, LOT 23110306; exp. 2 November 2026). Each pouch consisted of one side made from ARJO medical dialysis paper (60 g/70 g) and the other from PET/CCP transparent laminate film. The pouches were printed with ISO11140-1-compliant, non-toxic, and environmentally friendly ink.

### 2.2. Sterilization Process

All screws in Groups 1 and 2 were autoclaved by a sterile processing technician using a universal B cycle at 210 kPa and 134 °C for 50 min in the same autoclave (Phoenix blue printer, GUARISCO MEDICAL STERILIZER s.r.l., 222071 Cadorago (CO), Italy). The appropriate color change in chemical indicators was verified, and all pouches were checked before storage. The proper temperature, pressure, and time were adhered to, and all physical indicators were evaluated during every cycle. A thermal printer integrated into the sterilizer generated a printed record for every cycle, allowing for the immediate verification and archiving of sterilization conditions. Biological indicators were evaluated monthly as part of a quality assurance program (quality management system in compliance with UNI ENI ISO 9001:2015, certificate number 0039.2024-13).

The sterilization date and the future opening date were identified on each pouch, as indicated in Table 1.

The sterilization date was the exact date when the screws were sterilized, while the future opening date, considering the expected storage period, corresponded to the moment in which the screws were opened and analyzed. On each pouch, the sterilization date and the future opening date were indicated with the day, month, and year, preceded by the abbreviations StD and FOD, respectively.

### 2.3. Storage Conditions

A steel cabinet within an ISO-6-classified operating room (quality management system in compliance with UNI ENI ISO 9001:2015, certificate number 0039.2024-13) was chosen as a suitable storage location, with no exposure to light, and daily cleaning and bacteriological tests were carried out periodically on the air filters and every surface in the room.

**Figure 1:**
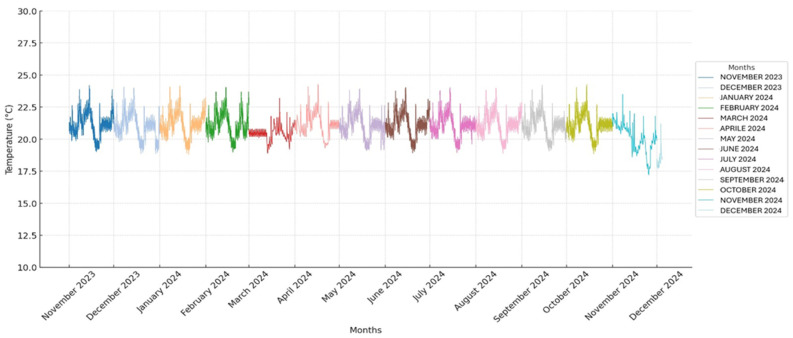
Temperature values recorded throughout the study period: the x-axis represents time in months, with each month indicated by a specific color, as shown in the legend, and the y-axis indicates temperature values in degrees Celsius.

**Figure 2:**
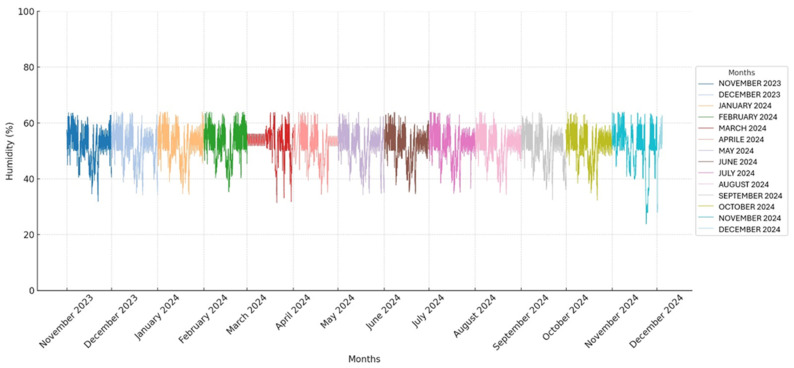
Humidity values recorded throughout the study period: the x-axis represents time in months, with each month indicated by a specific color, as shown in the legend, and the y-axis indicates humidity values in percentage.

The temperature and humidity in the storage room were monitored using a thermohygrometer to detect abrupt fluctuations that could interfere with the usual storage of the pouches. All obtained temperature and humidity values are presented in two graphs (Figure 1 and Figure 2).

Following the guidelines of the sterilization material manufacturer, the screws were stored under controlled humidity and temperature conditions. The mean temperature and humidity are expressed as the mean ± standard deviation (SD).

### 2.4. Microbiological Testing

At 1, 7, 15, 30, 60, 182, and 390 days post-sterilization, 25 screws from both Group 1 and Group 2 were aseptically opened under a laminar flow hood in the Bacteriology Laboratory of the Department of Veterinary Medicine and Animal Production, University of Naples “Federico II”.

Control group screws (Group 3) were opened and tested one day prior to the first bacteriological analysis of screws stored for one day.

All pouches were transported to the Bacteriology Laboratory using a closed box that was cleaned daily. The laboratory is on the second floor of the same building as the storage room.

The sterile technique, under a laminar flow hood, involved the opening of the pouches by an experienced operator and the removal of the screws from the pouch using sterile surgical forceps; a new pair of sterile forceps and new pair of sterile gloves were used for any new screws. Once opened, the collected screws were inoculated in brain heart infusion broth (Liofilchem, Teramo, Italy) and incubated aerobically at 37 °C for 24 h.

After the overnight incubation, broth turbidity was assessed, and when necessary, a broth sub-cultivation was performed on Columbia blood agar base plates (Liofilchem, Teramo, Italy), a medium for the isolation of both Gram-positive and Gram-negative bacteria, and on Sabouraud Dextrose agar (SDA) plates (Liofilchelm, Teramo, Italy) for yeast detection. The identification of recovered isolates was performed by Matrix-Assisted Laser Desorption/Ionization–Time-Of-Flight Mass Spectrometry (MALDI–TOF MS) (Bruker Daltonics GmbH, 28359 Bremen, Germany). Score values of less than 1.7 were considered unreliable for identification; scores between 1.7 and 1.99 suggested a probable identification at the genus level, while scores of 2.0 or higher indicated a certain genus identification and a probable-to-highly probable species-level identification.

### 2.5. Statistical Analysis

Data concerning temperature and humidity were collected and recorded using spreadsheet software (Microsoft^®^ Excel^®^ 2011, Microsoft Corporation). Continuous variables were expressed as the mean ± SD.

Two tables of 2 × 2 were constructed to compare the presence of bacterial growth (positive or negative): one table compared the sterilized and control groups, and the other compared Group 1 and Group 2.

Two Fisher’s Exact Tests were performed: one between the sterilized groups and the control group, and one between the two sterilized groups. In all analyses, *p* < 0.05 was considered statistically significant.

To assess whether the temperature and humidity remained stable throughout the study, the coefficient of variation (CV) was calculated.

## 3. Results

Over 390 days, from 10 November 2023, to 8 December 2024, a total of 600 pouches were used in this study, which was conducted at the VTH of the Department of Veterinary Medicine and Animal Production. The sterilization date and the future opening date of the pouches were respected for each stated time point, in accordance with the experimental setup. No envelopes were found to be altered or damaged; chemical indicators changed their color correctly, confirming the correct sterilization process. Physical indicators revealed that all the processes were correct. Biological indicators are checked monthly, and the effectiveness of the sterilization process is confirmed by demonstrating the inactivation of highly resistant bacterial spores (*Geobacillus stearothermophilus*).

A total of 37,440 temperature and humidity detections were analyzed by a thermo-hygrometer that recorded the values every 15 min for 390 days. All recorded temperature and humidity values are presented in two graphs (Figure 1 and Figure 2).

The average temperature and humidity readings in the storage room were 20.9 °C (±0.97 SD) and 52.1% (±5.6 SD), ranging from 17.2° C to 24.3 °C and 23.8% to 63.9%, respectively.

The CV for temperature was 4.6%, and the CV for humidity was 10.8%.

The bacteriological tests on the steel storage cabinet every 30 days showed a bacterial growth of less than five colony-forming units on 24 cm^2^; no microorganisms indicative of environmental microbiological contamination were found on any of the surfaces tested. These tests were conducted as part of the hospital’s quality control measures (quality management system in compliance with UNI ENI ISO 9001:2015, certificate number 0039.2024-13).

The microbiological investigations of the screws, Groups 1 and 2, after the overnight incubation, gave negative results for both bacteria and yeasts up to the 182-day storage time. After 390 days of storage, bacterial growth was observed in one screw from Group 1 (single pouch), which tested positive for *Klebsiella pneumoniae* (MALDI–TOF MS score identification: 2.20), recovered as a monoculture, while no growth was detected in Group 2 (double pouch). No yeasts were isolated from the screws of both groups.

The results were reported for Groups 1 and 2 as being “negative” and “positive” for bacterial growth, as described in Table 2.

For the control group (Group 3), two screws showed positive results, with visible turbidity in the brain heart infusion tubes, indicating bacterial growth. Subsequent culturing was performed on Columbia blood agar plates to determine the presence of Gram-positive and Gram-negative bacteria and on SDA for yeast isolation. *Staphylococcus epidermidis* (MALDI–TOF MS score identification: 2.34) was isolated and identified from one sample, while *Bacillus cereus* (MALDI–TOF MS score identification: 2.05) was isolated from a different one. Both bacterial isolates were detected as pure cultures. The two bacteria-positive samples did not yield any yeast isolates.

To perform the two Fisher’s Exact Tests, two 2 × 2 tables were created: the first illustrates the bacterial growth of screws between the control group and the sterilized groups (Table 3), while the second focuses on the bacterial growth between the single-pouch and double-pouch groups (Table 4).

The Fisher’s Exact Test between the control and sterilized groups yields a *p*-value of 0.042 (*p* = 0.042).

The Fisher’s Exact Test between Group 1 and Group 2 yields a *p*-value of 1.0 (*p* = 1.0).

## 4. Discussion

The study aimed to assess the shelf life of surgical screws stored under controlled conditions and compare two different packaging methods, namely using a single and double self-sealing pouch. Furthermore, this study also validated the reliability of the sterilization process performed at the VTH.

The lack of specific guidelines about the storage time of sterilized surgical instruments, especially in veterinary practice, represents a problem; without defined standards, it is possible to extend surgical instruments’ shelf life excessively, potentially compromising patient safety, and increasing costs, management time, and environmental pollution.

Indeed, different studies have revealed that the shelf life of packaging material is mainly related to the environmental conditions of the storage location and the packaging material used [5,6,17]. Although current studies have emphasized that sterility is primarily event-related [6,18], expiration dates are still assigned to surgical materials to ensure patient safety and minimize the risk of infections.

These expiration dates may often be based on conservative criteria and do not always accurately reflect the actual duration for which sterility can be maintained under controlled conditions.

Several studies have indicated that the storage environment might be a more important factor than the type of packaging material in maintaining sterility [19,20]. While the packaging method is reproducible, it is not possible to reproduce identical environmental conditions; so, to the best of our knowledge, it becomes difficult to know the exact expiry date of sterile surgical material unless a self-protocol is carried out. Furthermore, storing surgical materials under the same environmental conditions is essential for comparing two different sterilization methods.

Paper/plastic peel pouches are widely used as packaging materials for steam sterilization in an autoclave due to their convenience, content visibility, and effectiveness. They should be used in accordance with the manufacturers’ written instructions [14]; specifically, self-sealing pouches do not require a heat-sealing machine, and based on our experience, they are easy to use with a short packaging time.

Several studies have analyzed the shelf life of sterile materials packaged with different methods [15,16,18]. However, there has been no study evaluating the shelf life of surgical instruments with complex surfaces that are packaged with single or double paper/plastic self-sealing pouches and sterilized by steam sterilization. The selection of orthopedic screws for this study was based on two key factors: their daily use in the operating rooms of the VTH of Naples and their complex surface, which can easily trap dust or organic material [21,22].

The control group (Group 3) confirmed the reliability of the bacterial detection method, with two screws showing turbidity upon examination, indicative of bacterial growth. Specifically, *Bacillus cereus* and *Staphylococcus epidermidis* were the bacterial species isolated in monocultures from two different samples. This outcome, consistent with the results of other studies performed on packaged surgical instruments [15,16], validated that the testing process used for the study can identify bacterial contamination when it is present, supporting the accuracy of the sterility assessments for Groups 1 and 2.

The CV was calculated to assess whether the temperature and humidity remained stable throughout the study. The CV for temperature was found to be 4.6%, indicating a relatively low level of variability and suggesting that the temperature remained stable during the observation period. In contrast, the CV for humidity was 10.8%, showing a higher degree of variability and indicating that the humidity levels fluctuated more throughout the study.

Fisher’s Exact Test was selected for statistical analysis. This method was applied in two instances: to compare the control group with the sterilized groups and to assess the statistical significance between the single-pouch and double-pouch groups. Fisher’s Exact Test was chosen due to the sample size and its ability to analyze groups with unequal sample sizes.

According to our results, Fisher’s Exact Test between non-sterilized screws and sterilized screws found a *p*-value of 0.042, so it is possible to conclude that there is a statistically significant difference in relation to bacterial growth of the tested groups. No statistical difference was found between Group 1 and Group 2 (*p* = 1.0); these findings do not fully align with some studies evaluating single versus double packaging methods, which have generally reported no significant advantages in using double packaging over single packaging [15,16]. Differences between our findings and previous studies may reflect methodological variations, including the use of different pouch materials and storage environments. Unlike studies conducted in human hospital settings, our investigation was performed under controlled ISO 6 conditions in a veterinary facility, which may involve different microbial exposure levels and contamination risk.

To conduct a time-based study, in addition to closely monitoring environmental conditions, it was ensured that the main adverse effects that could compromise sterility did not occur.

The main adverse events that could potentially compromise the sterility of the surgical instruments include an insufficient number of instruments in the package, worn-out instruments with poor performance, unclear or altered markings, incorrect dates, improper packaging materials, missing internal chemical indicators, and the presence of stains or holes in the cloth wrapping material [23].

Throughout the entire process of sterilization, storage, and opening of the pouches, no incidents were observed that could have damaged or compromised the integrity of the packaging. The sterile processing technician, who carefully handled the pouches weekly, checked their integrity and condition each day, ensuring that the sterility of the instruments remained intact under the conditions of this study.

Consequently, it is reasonable to assume that our study accurately evaluates the shelf life of the surgical materials purely based on time, without the influence of any observed adverse events.

Despite no statistical significance being found between the two groups, a single positive case in the single-pouch group may have clinical relevance, highlighting the need for further research.

A single positive result also suggests that, despite the absence of observed adverse events and the modern preference for event-related sterility over time-related approaches, a prolonged storage time might represent an event that could potentially compromise sterility under controlled environmental conditions, although further studies are needed to confirm this trend.

Some limitations were encountered in performing this study. One of the limitations was the observation period, which lasted 390 days; this might not have been sufficient to observe long-term changes in the sterility and degradation of the sterilized materials. The microbial growth was assessed only as presence or absence, evaluating the broth turbidity followed by plating on selected solid media, in order to identify the isolated microbial species. Extended observation periods could yield more comprehensive data regarding the shelf life of surgical instruments and their susceptibility to environmental factors over time.

Furthermore, the findings of this study may be challenging to replicate in different veterinary hospital environments due to variability in the storage conditions, climate, and handling practices. Each veterinary hospital operates under unique circumstances, which can significantly influence the outcomes related to sterilization and packaging efficacy.

Lastly, the study focused solely on a single type of packaging method—paper/plastic self-sealing pouches. While these pouches are commonly used and practical, the results may not apply to other packaging materials or configurations.

## 5. Conclusions

In conclusion, double pouches provided a shelf life of at least 390 days, and no statistical difference in contamination was observed between the groups. However, the single contaminated screw in the single-pouch group highlights a potential clinical consideration.

## Figures and Tables

**Table 1:** Storage time, number of screws opened, sterilization date, and future opening date.

Storage Time	1 Day	7 Days	15 Days	30 Days	60 Days	182 Days	390 Days
Screws Groups 1 and 2	25 for each group	25 for each group	25 for each group	25 for each group	25 for each group	25 for each group	25 for each group
Sterilization date	21 February 2024	21 February 2024	22 February 2024	29 February 2024	21 February 2024	21 March 2024	10 November 2023
Futuring opening date	22 February 2024	28 February 2024	8 March 2024	30 March 2024	21 April 2024	19 September 2024	4 December 2024

**Table 2:** Storage time (in days) and bacterial growth divided by Group 1 and Group 2.

Storage Time	Group 1—Bacterial Growth	Group 2—Bacterial Growth
1 day	Negative	Negative
7 days	Negative	Negative
15 days	Negative	Negative
30 days	Negative	Negative
60 days	Negative	Negative
182 days	Negative	Negative
390 days	Positive (1/25 screws, 4%)	Negative

**Table 3:** Distribution of positive and negative outcomes among screws in the sterilized groups (Groups 1 and 2) and non-sterile control group (Group 3).

	Positive (Bacterial Growth)	Negative (No Bacterial Growth)
Sterilized screws	1	349
Non-sterilized screws	2	48

**Table 4:** Distribution of positive and negative outcomes among screws in the two sterilized groups.

	Positive (Bacterial Growth)	Negative (No Bacterial Growth)
Group 1	1	174
Group 2	0	175

## Data Availability

The original contributions presented in this study are included in the article/Supplementary Materials.

## Supplementary Materials

The following supporting information can be downloaded at: https://www.mdpi.com/article/10.3390/vetsci12060564/s1, Table S1. MALDI–TOF MS identification of bacteria from sterilized and non-sterilized surgical screws.

## Abbreviations

The following abbreviations are used in this manuscript:

SSIs	Surgical-Site Infections
VTH	Veterinary teaching hospital
SD	Standard deviation
CV	Coefficient of variability
