# Supplementary material for: A Shelf-Life Assessment of Sterilized Surgical Instruments Stored Under Controlled Conditions: A Comparative Study of a Single vs. Double Self-Sealing Pouch
Source: Vet Sci. 2025 Jun 9;12(6):564. doi: 10.3390/vetsci12060564 (PMC12197567; doi:10.3390/vetsci12060564)
Supplement: Supplementary file 1 [file vetsci-12-00564-s001.zip › vetsci-3616285-supplementary.pdf]

TABLE S1. MALDI-TOF MS identification of bacteria from sterilized and non-sterilized surgical screws.

Samples	Bacterial species	MALDI-TOF MS (log)score
n. 1 sterilized screw	*Klebsiella pneumoniae*	2.20
n. 1 non-sterilized screw	*Bacillus cereus*	2.05
n. 1 non-sterilized screw	*Staphylococcus epidermidis*	2.34
